# Homeowner perceptions and responses to buffelgrass invasion risk in the Tucson, Arizona Wildland-Urban Interface

**DOI:** 10.1016/j.heliyon.2021.e07040

**Published:** 2021-05-27

**Authors:** Abigail F. Plecki, Kofi Akamani, John W. Groninger, Jacob C. Brenner, Karla L. Gage

**Affiliations:** a Forestry Program, Southern Illinois University, Carbondale, IL, USA; b Department of Environmental Studies and Sciences, Ithaca College, Ithaca, NY, USA; c Plant, Soils, and Agricultural Systems/Plant Biology Program, Southern Illinois University, Carbondale, IL, USA

**Keywords:** Biological invasion, Capital assets, Community resilience, Institutions, Invasive species, Urbanization

## Abstract

In this study, we aimed to analyze homeowners' level of awareness and perceived risk about buffelgrass invasion in the Tucson, Arizona Wildland-Urban Interface (WUI), as well as the factors influencing their participation in buffelgrass control and fire risk mitigation efforts. Data for the study were generated through the administration of an online survey among 117 members of Home Owner Associations (HOAs) in the Tucson WUI. The results showed that the overwhelming majority of respondents were aware of buffelgrass, but their knowledge about buffelgrass control mechanisms appeared to be limited. Respondents also more frequently expressed concern about the risks posed by buffelgrass invasion to general targets, such as the Sonoran Desert ecosystem, native plants and wildlife than risks to their private property and neighborhoods. The results also showed that the level of involvement in HOAs, and leadership in HOAs had significant positive effects on homeowners' participation in buffelgrass control efforts. Homeowners' duration of residence also had a significant negative effect on participation in buffelgrass control efforts, suggesting that newcomers may be more involved than long-term residents. Similarly, the number of months respondents spent in Tucson per year had a negative effect on the number of hours spent on buffelgrass control efforts. Respondents’ perceived risk about buffelgrass invasion also had a positive effect on the hours spent on buffelgrass control as well as their level of involvement in fire risk mitigation efforts. These results highlight the importance of local institutions and community heterogeneity in social responses to threats in WUI communities. Policies aimed at building the resilience of WUI communities need to account for their complexity as coupled social-ecological systems.

## 1 Introduction

Since the 1970s, urban deconcentration has been occurring in the United States (Pacione 2009), including suburbanization, exurbanization, and the expansion of the “wildland-urban interface” (WUI) (Hammer et al., 2009). The WUI has been conceptually defined as the area where human settlements meet or overlap with wildlands (Steward et al., 2007; Bar-Massada et al., 2014), and two categories have been identified. Intermix WUI refers to areas where housing development intermingles with wildland vegetation; and interface WUI refers to areas where human settlements abut natural landscapes (Radeloff et al., 2005; Bar-Massada et al., 2014). Changes in the geographic distribution of the WUI population, including increases in low-density housing and encroachment on natural areas (Hammer et al., 2009; Radeloff et al., 2005), are largely driven by amenity migration, the movement of people to places with desirable natural and cultural features (Rudzitis 1999; Charnley et al., 2008). Some have argued that WUI environments constitute unique social-ecological systems (Bar-Massada et al., 2014).

The wildfire risks posed to human lives and property in WUI communities have received significant research and policy attention in recent years (Winter and Fried 2000; Gill and Stephens 2009; Mell et al., 2010; Calkin et al., 2014). However, residential development at the WUI also presents a number of ecological threats, including habitat loss and fragmentation, biodiversity decline and threats to wildlife populations (Radeloff et al., 2005). According to Bar-Massada et al. (2014), residential development in the WUI can facilitate exotic species introduction through enhanced availability of propagules (from residential gardens, horticultural landscapes and garden waste), the formation of new habitat edges, and the transport of propagules across those edges. While biological invasions have received considerable research attention in recent years (Kalnicky et al., 2019), a limited number of studies have examined this problem in WUI communities (Bar-Massada et al., 2014). Importantly, relatively little has been published on the interactions between wildfire and biological invasions. More work is needed in this area to better understand the risks posed to both human communities and valued ecosystems (Brenner and Franklin 2017).

Communities at the WUI exhibit features of complex social-ecological systems (Folke et al., 2010), including path-dependency, cross-scale interactions, heterogeneity, and surprise (Paveglio et al., 2009; Hammer et al., 2009; Bar-Massada et al., 2014). When WUI communities are conceptualized as complex social-ecological systems (Bar-Massada et al., 2014), the community resilience perspective provides a powerful tool for analyzing the dynamic interactions between the social and ecological components of WUI communities over multiple spatial and temporal scales. Community resilience refers to the ability of communities to respond to drivers of change in a manner that results in the maintenance or enhancement of community well-being (Akamani 2012). Drawing from the broader resilience literature, the concept of community resilience assumes that communities across the rural-urban continuum are constantly exposed to various drivers of change, including culture, politics and policy, demography, economics, technology, and natural ecosystem dynamics to which they must adapt (Berks and Ross 2013; Akamani and Hall 2015). The ability of communities to respond to these drivers of change is determined by the stock of community capital assets, such as social capital, human capital, economic capital, physical capital, and natural capital, as well as the availability of effective community institutions and organizations (Flint 2010; Akamani 2012).

Capital assets are critical determinants of the level of exposure of communities to various threats, as well as the ability of community members to absorb such threats (Magis 2010; Akamani and Hall 2019). Several studies on WUI fire risk show that vulnerable communities with fewer capital assets have lower ability to engage in mitigation programs (Mercer and Prestemon 2005; Gaither et al., 2011). Local institutions and organizations also play essential roles in building community resilience and reducing vulnerability by enhancing access to information and incentives, as well as resources and opportunities (Akamani et al., 2015). All of these influence social responses to change events (Hendriks and Stokmans 2020).

The role of institutions and organizations in enhancing social responses to drivers of change in WUI communities has not received adequate research attention. Access to institutions and resources is likely to be socially differentiated, which would hypothetically give rise to differential abilities of WUI community members to respond to drivers of change. Our study addressed this research gap by focusing on community responses to the coupled threats of plant invasion and wildfire at a WUI site in Tucson, Arizona, USA. Our purpose was to examine some of the institutional and organizational factors surrounding homeowners’ responses to these threats. Our research questions focused specifically on levels of awareness, perceived risks, and predictors of participation in invasive plant control and fire risk mitigation.

## 2 Description of study context

The city of Tucson is located in the arid Sonoran Desert region of the American Southwest. Tucson is the second largest city in Arizona with an estimated population of 548,073 as of 2019 (US Census Bureau 2020). Some of Tucson's most expensive homes and fastest growing neighborhoods are located within the city's WUI. The alluvial slopes and foothills of the surrounding mountain ranges are among the most desirable locations for new housing development, and these areas are also habitat for native desert plants and wildlife. Tucson's rapid growth in recent decades has encroached on these highly valued desert ecosystems. The simultaneous introduction of non-native grasses, especially buffelgrass (*Cenchrus ciliaris*), has introduced wildfire to a previously fire-free landscape (Brooks and Chambers 2011; McDonald and McPherson 2011) and today poses a new threat to both desert ecosystems and the new inhabitants of the WUI.

Buffelgrass, which originates from the African Savannah, was introduced into the United States as a forage grass for livestock but has since become an invasive species that threatens to displace native plant communities in the Sonoran Desert and other parts of Arizona and New Mexico (USDA Forest Service 2014). Once buffelgrass becomes the dominant cover, it results in the decline of native species (Olsson et al., 2012). Fire professionals and natural area managers in the Tucson region have expressed concern that buffelgrass invasion is a growing threat to natural and built environments alike (Marshall et al., 2012; Cleetus and Mulik 2014). Fire and buffelgrass invasion appear to be mutually reinforcing, with increasing fire frequency and intensity raising the likelihood for permanent transformation of the landscape to a new, savannah-like state (Brenner 2010; Brooks and Chambers 2011). According to the Pima County Wildfire Protection Plan, prepared in 2013, there are a total of 1,579,699 acres, with a reported 121,511 WUI acres categorized as high risk for wildfire. Climate change projections for the Sonoran Desert include increased drought frequency and intensity, which further exacerbate fire risk and the potential for permanent changes in vegetation cover (Archer and Predick 2008).

Our study focused on the Tucson WUI case to better understand how homeowners perceive and respond to these emerging threats. A better understanding, in particular, of how homeowners interact with community institutions can help inform WUI policy for addressing emerging challenges.

## 3 Methods

This study was approved by the Institutional Review Board at Southern Illinois University Carbondale. The purpose of the study was to assess the perceptions and responses of Tucson homeowners to threats posed by buffelgrass invasion. Our population of interest was home owners residing within the Tucson WUI and our sampling frame comprised members of homeowner associations (HOAs) within the Tucson WUI. A homeowner association refers to an organization of homeowners of “residential developments consisting of several parcels of similar single-family, detached housing and, in many cases, that provide members with various goods and services such as street maintenance, snow removal, trash collection, and security patrol” (Groves 2006). Information gathered from the website of the City of Tucson indicated that there are a total of 146 HOAs in the city, out of which 38 of them were chosen for this study based on their location within the city's WUI, as well as the consent of their leadership to participate in the study. Respondents were recruited using self-selection sampling, a sampling technique that is used when the researcher wants members of a population of interest to choose to participate in the study on their own accord (Mujere 2006). The researchers identified the contact information of the representatives of all HOAs within the Tucson WUI by visiting the websites of each organization and these representatives were contacted to solicit the involvement of their members in the study. An email containing a link to the SurveyMonkey website through which the questionnaire was administered was then sent to the representatives of the 38 selected HOAs, who in turn forwarded the email to their members. The survey was administered between June 2017 and August 2017, and a total of 117 useable responses were received. Survey respondents represented 30 HOAs located within the Tucson WUI, out of the total of 38 HOAs whose members were invited to participate in the study.

The survey questionnaire was designed to measure a number of constructs, including the socio-demographic characteristics of respondents, involvement and leadership in HOAs, knowledge and risk perception about buffelgrass invasion, and participation in buffelgrass control and fire risk mitigation efforts. Socio-demographic characteristics on which data were collected in the study included the number of months per year respondents resided in Tucson, duration of residence, level of education, and motivation for residing in Tucson. To measure respondents' level of involvement in the neighborhood HOAs, a list of five HOA activities were included in the questionnaire, to which respondents were asked to rate their level of involvement on a three-point Likert-type scale (1 = never, 2 = sometimes, and 3 = always). The leadership status of respondents in their HOAs was also measured as a dichotomous variable (1 = yes, and 0 = no). Respondents' level of knowledge on buffelgrass was assessed using several measures, including sources of information about buffelgrass, length of time since respondent first heard about buffelgrass, knowledge of buffelgrass seed dispersal mechanisms, as well as methods for buffelgrass control on public lands and private property. Images of various plant species were also included in the questionnaire and respondents were asked to correctly identify those that represented buffelgrass. To assess respondents' perceived risk about buffelgrass invasion within the Tucson WUI, a question containing a list of 11 susceptible features perceived to be at risk, ranging from the individual to the Sonoran Desert was included in the questionnaire and respondents were asked to choose all that applied to them. Two questions were used to assess homeowners' involvement in buffelgrass control efforts. One contained a list of seven statements on various buffelgrass control activities and respondents were asked to rate their level of involvement on a three-point Likert-type scale (1 = never involved; 2 = sometimes involved; and 3 = always involved). Another question asked about the number of hours respondents had devoted to buffelgrass control efforts, and respondents were asked to choose from one of four response options. Finally, homeowners’ involvement in fire risk mitigation efforts was captured using a list of six statements on various fire risk mitigation activities to which respondents were asked to rate their involvement (1 = yes, and 0 = no).

Following the data collection process, the data were organized by calculating composite indices for relevant constructs that were measured with multiple items. Mean scores were computed to derive the indices for homeowner involvement in HOA activities and homeowner involvement in buffelgrass control efforts. Composite indices for homeowners' perceived risk and involvement in fire risk mitigation were derived using the sum of responses. Similarly, homeowner knowledge about buffelgrass was derived using the sum of correct responses to questions on buffelgrass identification, seed dispersal mechanisms, and the geographic distribution of the species. Following this, the data were analyzed using descriptive statistics to assess homeowners' level of awareness and perceived risk. Multiple regression analysis was also used to analyze the predictors of homeowners’ participation in buffelgrass control and fire risk mitigation efforts, and the results were interpreted based on significant (alpha = 0.10). Given the limited research on the responses of WUI communities to biological threats, the choice of our relatively low confidence level of 90% has support in the literature (e.g. Skipper et al., 1967; Michaels 2017; Miller and Ulrich 2019) and was intended to enable us to explore the explanatory utility of a wider range of predictor variables that could contribute to theory building in the future. We ran two separate multiple regression models to test the predictors of homeowner participation in buffelgrass control efforts using the number of hours spent on buffelgrass control efforts and a composite index for participation in buffelgrass control efforts as the outcome variables. We also ran a third regression model using a composite index for homeowner participation in fire risk mitigation efforts as the outcome variable. Drawing partly from the community resilience literature and from the broader social science literature on social responses to drivers of change (e.g. Akamani 2012; Gorddard et al., 2016; Bouman et al., 2021), our regression models were based on the hypothesis that homeowner responses to buffelgrass invasion and fire risk would be predicted by their involvement in HOAs (positive), their leadership status in HOAs (positive), level of knowledge about buffelgrass (positive), level of concern about buffelgrass risk (positive), as well as their socio-demographic attributes, such as level of education (positive), duration of residence (positive), number of months spent in Tucson per year (positive), and level of involvement in outdoor recreational activities (positive).

## 4 Results

### 4.1 Participants’ profile

The duration of residence of respondents in Tucson varied widely, with some respondents having lived there since the 1930s while others reporting having moved there within the last year. A sizable proportion of respondents reported moving to Tucson in recent decades, with 48.7% moving there after the year 1990. As much as 97.4% reported living in Tucson between seven to twelve months each year, while the rest of them reported that they lived in Tucson between one and six months out of the year. Respondents' level of education was generally high, with 88.7% having obtained a bachelors’ degree or higher.

During the survey, respondents were also asked about the factors that influenced their decision to live in Tucson. The results, presented in Table 1, shows that the top three factors for homeowners living in Tucson are the natural desert landscape (58.3%), the warm climate (50.4%), and employment (47.8%). Overall, non-economic factors, including natural and cultural amenities appear to play an important role in why people continue to live in Tucson, and why others choose to migrate there.

**Table 1:** Importance of community attributes in respondents’ decision to live in Tucson.

Factor	Frequency (n = 115)	Response (%)
The natural desert environment	67	58.3
Warm climate	58	50.4
Employment	55	47.8
Scenic views	54	47.0
“Wildland” outdoor recreation	52	45.2
Dry climate	43	37.7
Family roots in region	28	24.3
Vibrant community	26	22.6
College or university	26	22.6
Family currently in the region	24	20.9
Good place to raise family	23	20.0
“Urban” outdoor recreation	21	18.3
Cultural events or attractions	21	18.3
Retirement	19	16.5
Health or wellness	19	16.5
Safe communities	7	6.1
Good schools for children	4	3.5

Data on homeowners’ involvement in their HOAs, as well as their leadership in these local organizations were also analyzed and the results showed that the majority of respondents reported participating in HOA activities. For instance, 30.4% reported always attending HOA meetings; 37% reported sometimes attended their HOA meetings; and 32.1% reported never attending their HOA meetings. Membership in HOA leadership positions was generally low, with only 27.6% reporting holding a leadership position now or having held it in the past, while the majority of them reported having never held an HOA leadership position.

### 4.2 Homeowners’ level of knowledge and risk perception about buffelgrass invasion

The analysis of data on respondent's level of knowledge on buffelgrass showed that buffelgrass was known to nearly all respondents. As much as 95.7% reported having heard about buffelgrass, with 66.7% having heard about buffelgrass over 10 years prior to the survey. Only 4.3% of respondents had never heard about buffelgrass prior to the survey. However, respondents displayed an incomplete understanding of buffelgrass life history traits and control mechanisms. For instance, when asked to identify potential dispersal mechanisms, respondents identified wind most frequently (96.7%), followed by humans or animals (78.2%), water (60.9%), mowing (55.4%), and fire (29.4%). Respondents also most frequently identified manual removal as the most effective method for buffelgrass control on both private (87.5%) and public property (77.5%) (Table 2). Herbicide was the second most frequently identified buffelgrass control method on both public (21.6%) and private lands (9.8%).

**Table 2:** Homeowner perceptions of efficacy of buffelgrass control methods on private and public land.

Variable	Frequency	Response (%)
*Most Effective Control Method on Private Property*	(n = 112)	
Chemical herbicide	11	9.8
Mowing	1	0.9
Manual removal	98	87.5
Buffelgrass does not need to be controlled	2	1.8
*Most Effective Control Method on Public Lands*	(n = 111)	
Chemical herbicide	24	21.6
Manual removal	86	77.5
Prescribed burn	1	0.9
Buffelgrass does not need to be controlled	0	0.0

Results on homeowners’ risk perceptions about buffelgrass invasion showed a focus on elements of the natural landscape, such as native plants (95.5%), esthetic features of the Sonoran Desert (93.8%), and wildlife (75%) (Table 3). Roughly half of the respondents (50.9%) reported that they felt that buffelgrass invasion puts their home at risk. Less than half of survey respondents (34.8%) reported that the City of Tucson was at risk of buffelgrass invasion.

**Table 3:** Respondents’ perceptions of targets considered to be at risk.

Variable	Frequency (n = 112)	Response (%)
Native plants	107	95.5
Sonoran Desert aesthetics	105	93.8
Wildlife	84	75.0
My neighborhood	64	57.1
My home	57	50.9
Property values	50	44.6
Favorite outdoor recreation	48	42.9
Myself	39	34.8
City of Tucson	39	34.8
Soil salinity	31	27.7
My job	15	13.4

### 4.3 Homeowner involvement in buffelgrass control

Data on homeowners’ involvement in buffelgrass control efforts were also gathered by asking survey respondents to rate their level of involvement in a list of buffelgrass control activities. The results, reported in Table 4, show that the majority of respondents (76.9%) reported being sometimes or always involved in the control of buffelgrass on their property. Another 54.7% of respondents reported being involved in buffelgrass control efforts in their neighborhoods. However, the overwhelming majority of respondents reported never being involved in the rest of the buffelgrass control activities, which include making donations to buffelgrass causes and being involved in buffelgrass organizations.

**Table 4:** Homeowner involvement in buffelgrass control.

Variable	Frequency (n = 117)	Response (%)
*Controlling on my property*		
Always	79	67.5
Sometimes	11	9.4
Never	27	23.1
*Controlling in my neighborhood*		
Always	18	15.4
Sometimes	46	39.3
Never	53	45.3
*Controlling with Tucson Clean and Beautiful*		
Always	0	0.0
Sometimes	14	12.0
Never	103	88.0
*Controlling with Sonoran Desert Weedwhackers*		
Always	1	0.9
Sometimes	10	8.5
Never	106	90.6
*Attending the annual Beat Back Buffelgrass Day*		
Always	8	6.8
Sometimes	14	12.0
Never	95	81.2
*Involvement in another buffelgrass organization*		
Always	8	6.8
Sometimes	10	8.5
Never	99	84.6
*Donating to buffelgrass causes*		
Always	0	0.0
Sometimes	16	13.8
Never	100	86.2

### 4.4 Homeowner involvement in fire risk mitigation

To generate data on homeowners’ involvement in fire risk mitigation efforts, survey respondents were also asked to indicate whether or not they have been involved in a number of fire risk mitigation activities. The results in Table 5 show that the majority of respondents reported taking appropriate actions that reduced fire risk to their homes.

**Table 5:** Fire Risk mitigation participation rates by respondents.

Practice	Participation Rate (%)
Fence made up of non- combustible material	87
Chimney screen	71
Maintain free of leaf litter	71
Trees at least 6 ft from house	58
Buffer between grass and dense vegetation and home	91
Roof and gutters free of litter	91

### 4.5 Factors influencing homeowner participation in buffelgrass control

The results of multiple regression analysis on the predictors of homeowners' participation in various buffelgrass control activities showed that HOA involvement and HOA leadership each had a statistically significant positive effect on respondents’ participation in buffelgrass control while duration of residence had a negative effect on participation in buffelgrass control (Table 6). In contrast, level of knowledge about buffelgrass, perceived buffelgrass risks, and socio-demographic attributes, such as level of education, number of months spent per year in Tucson, and level of involvement in outdoor recreational activities did not predict participation. Among those who participated in buffelgrass control, perceived buffelgrass risks had a positive effect on the number of hours spent on buffelgrass control, while the number of months spent in Tucson had a negative effect on hours spent on those efforts (Table 7).

**Table 6:** Regression results for predictors of buffelgrass control involvement.

Independent Variables	*B*	*SE B*	β	*p* value
HOA Involvement	0.26	0.11	0.28	0.03
HOA Leadership	1.11	0.36	0.37	0.002
Level of Buffelgrass Knowledge	0.04	0.04	0.12	0.26
Highest Degree	-0.18	0.16	-0.12	0.27
Duration of residence	-0.02	0.01	-0.27	0.01
Months in Tucson	-0.09	0.12	-0.08	0.47
Outdoor Recreation	0.03	0.02	0.15	0.17
Perceived risk	0.00	0.00	0.12	0.27
R-squared	0.20			
Adjusted R-squared	0.12			
*F* statistic	2.41

**Table 7:** Regression Results for predictors of total hours of buffelgrass control.

Independent Variables	*B*	*SE B*	β	*p* value
HOA Involvement	0.03	0.10	0.04	0.74
HOA Leadership	-0.30	0.31	-0.11	0.34
Level of Buffelgrass Knowledge	-0.02	0.03	-0.06	0.54
Highest Degree	0.14	0.14	0.10	0.34
Duration of Residence	0.00	0.01	0.03	0.75
Months in Tucson	-0.18	0.11	-0.17	0.10
Outdoor Recreation	0.01	0.02	0.03	0.52
Perceived Risk	0.01	0.00	0.42	0.00
R-squared	0.24			
Adjusted R-squared	0.16			
*F* statistic	3.00			

### 4.6 Factors influencing homeowner participation in fire risk mitigation efforts

Results of regression analysis on the predictors of homeowners’ involvement in fire risk mitigation activities showed that the model explained 18% of the variance in the dependent variable (F = 1.81, p < 0.1). It was found that perceived risk had a statistically significant positive effect on homeowners’ participation in fire risk mitigation actions (β = 0.28, p < 0.01). None of the other independent variables included in the model was statistically significant.

## 5 Discussion

In this study, we used survey data to analyze the level of awareness and risk perception about buffelgrass invasion among homeowners in the Tucson, Arizona WUI, as well as their involvement in buffelgrass invasion control and fire risk mitigation initiatives. The results of the survey suggest that although the majority of respondents (95.7%) had heard about buffelgrass prior to the survey, they did not seem to possess the requisite knowledge to effectively manage the species. While control efforts on private residential property focus on hand pulling, public lands rely heavily upon chemical control. Respondents' strong affinity for hand pulling on both public and private lands suggests that broadening buffelgrass control efforts may require the reconciliation of homeowner preferences and expectations of management outcomes. Respondents’ incomplete knowledge of buffelgrass dispersal is especially concerning as even an actively engaged population could unwittingly contribute to buffelgrass spread.

Data on respondents’ risk perception about buffelgrass invasion showed that homeowners widely held the perception that the surrounding wildland and native landscapes were at risk in the event of a buffelgrass invasion. The top three things considered by respondents to be at the most risk to buffelgrass invasion were native plants, Sonoran Desert esthetics, and wildlife. However, risk perception decreased when factors were within the city limits of Tucson or the private domain of the respondent. However, respondents also reported higher levels of involvement in buffelgrass control efforts on their private property and the neighborhood level than efforts targeting the larger Tucson community and the Sonoran Desert as a whole. Since the probability of exposure and the susceptibility to loss are key factors influencing risk (Calkin et al., 2014), the lower level of concern expressed by respondents about their private property and neighborhood could reflect a belief in their ability to protect those personal targets from harm. Indeed, the literature on risk perception shows that respondents tend to evaluate general risks higher than personal risks and this tendency for risk denial is strongly correlated with perceived control (Sjöberg 2000). Others have also identified the need to reduce feelings of fear and anxiety as potential explanations for the biased estimation of personal risks (Van der Pligt 1996).

HOA involvement, HOA leadership, and duration of residence were significant predictors of involvement in buffelgrass control efforts. The results showed that homeowners’ level of involvement in their HOAs had a positive effect on their levels of involvement in buffelgrass control efforts and this finding is consistent with the role of institutions and organizations in enhancing community resilience to threats (Agrawal and Perrin 2008; Akamani et al., 2015). As local institutions, HOAs may facilitate the sharing of information among homeowners, contribute to building relevant networks and social capital, and also facilitate the mobilization of critical resources in buffelgrass control efforts. The positive effect of HOA leadership on homeowner involvement in buffelgrass control efforts is also consistent with the literature (Larsen et al., 2018).

Homeowners who reported a longer duration of residence in Tucson were likely to report lowers level of involvement in buffelgrass control efforts. It is possible that newcomers to Tucson may be arriving with higher levels of environmental awareness (human capital), more financial resources (human capital), as well as other critical resources, including more free time to engage in community development efforts, such as buffelgrass control, than long-term residents. This finding on the potential divide between long-term residents and newcomers is consistent with the literature on amenity migration (Charnley et al., 2008; Hiner 2014). Amenity based migration is a widely-recognized driver of demographic change in the western United States and a potential source of conflict between newcomers who may hold pro-environmental values and longer-term residents who more often value natural resource extraction and agricultural interests. However, where effective community institutions exist to manage these social differences, amenity migrants can constitute an important source of human and economic capital, and therefore serve as an engine of community development.

Another regression model using the number of hours homeowners spent on buffelgrass control as an outcome variable showed that homeowners’ buffelgrass risk perception had a statistically significant positive effect on their participation in buffelgrass control efforts while the number of months homeowners spent in Tucson per year had a statistically significant negative effect on homeowner participation in buffelgrass control efforts. The negative effect of number of months of residence per year on participation in buffelgrass control is consistent with the results previously discussed on the effect of duration of residence. The positive effect of buffelgrass risk perception on participation in buffelgrass control efforts is also consistent with the literature that shows that perceptions of risk are often associated with preventive behavior (Van der Pligt 1996).

In another regression model on the predictors of homeowner participation in fire risk mitigation efforts, homeowners’ risk perception regarding buffelgrass threats also emerged as the only statistically significant positive predictor. These results further highlight the role of risk perception as a motivational factor in self-protective behaviors (Van der Pligt 1996).

It must, however, be noted that given the limited research that has been done on social responses to biological threats, such as buffelgrass invasion in WUI communities, our study was largely atheoretical and exploratory in nature. Our respondents were also selected from the members of HOAs using a non-ramdom sampling technique, thus limiting the generalizability of our findings to the larger Tucson population. Nonetheless, our results illustrate the potential utility of social science theory in understanding the complexity of WUI communities and their responses to various drivers of change. Future studies based on theories in sociology, social psychology and other relevant social science fields and using mixed methods approaches to collect data across a large number of WUI communities could yield valuable insights on how WUI communities act. Such theoretical insights will be essential in informing policies aimed at enhancing the sustainability and resilience of WUI communities and their surrounding ecosystems in the face of multiple drivers of change.

## 6 Conclusion

This study analyzed homeowners' knowledge and risk perceptions about buffelgrass invasion, as well as their involvement in buffelgrass control and fire risk mitigation efforts. The results suggest that membership and leadership in relevant organizations, such as HOAs have a positive influence on homeowner participation in buffelgrass control efforts. Homeowners' perceived buffelgrass risk also showed a positive effect on the number of hours homeowners spent on buffelgrass control, as well their participation in fire risk mitigation efforts. Moreover, homeowners who reported living in Tucson fewer months per year or reported a shorter duration of residence in Tucson were more likely to be involved in buffelgrass control efforts, suggesting that newcomers and short-term/seasonal residents may be more involved than long-term residents. These results may stem from differences in homeowners' level of knowledge about these environmental threats, and differences in resource endowments among others. In all, the results suggest that differences in homeowners' access to relevant institutions and organizations, as well as the capital assets that influence community resilience may help explain the differences in homeowners’ response to the buffelgrass risk and threats of fire to their private property, neighborhoods and communities.

These findings have important policy implications. First, a comprehensive approach to community capacity building, including the building of local institutional capacity, is needed to prepare WUI communities to deal with biological invasions, as well as other social and ecological problems associated with rapid urban expansion. Second, given the importance of risk perception as a predictor of homeowner responses to buffelgrass invasion and fire risk, an environmental education strategy aimed at broadening homeowners’ awareness about the risks posed by buffelgrass invasion and mechanisms for managing them may help increase participation in management efforts. Of particular importance is the need to deepen public understanding of the complex relationship between wildfire risk, native species management and other threats in WUI communities. In this way, buffelgrass management represents an opportunity to engage stakeholders with diverse motivations to find common ground in jointly addressing a safety and ecosystem health risk where rapid growth in human settlements appears to otherwise be inevitable.

## Declarations

### Author contribution statement

Abigail F. Plecki: Conceived and designed the experiments; Performed the experiments; Analyzed and interpreted the data; Contributed reagents, materials, analysis tools or data.

Kofi Akamani: Analyzed and interpreted the data; Contributed reagents, materials, analysis tools or data; Wrote the paper.

John W. Groninger: Conceived and designed the experiments; Performed the experiments; Analyzed and interpreted the data; Contributed reagents, materials, analysis tools or data; Wrote the paper.

Jacob C. Brenner: Conceived and designed the experiments; Performed the experiments; Contributed reagents, materials, analysis tools or data; Wrote the paper.

Karla L. Gage: Conceived and designed the experiments; Performed the experiments; Contributed reagents, materials, analysis tools or data.

### Funding statement

This work was supported by the USDA National Institute of Food and Agriculture, McIntire Stennis project 1020037.

### Data availability statement

Data included in article.

### Declaration of interests statement

The authors declare no conflict of interest.

### Additional information

No additional information is available for this paper.
